# Unintended Harm and Benefit of the Implantable Defibrillator in an Unfortunate 19-Year-Old Male: Featuring a Sequence of Rare Life-threatening Complications of Cardiac Procedures

**DOI:** 10.1016/s0972-6292(16)30650-7

**Published:** 2013-08-01

**Authors:** Amin Daoulah, Ali Ocheltree, Amir Lotfi, Sara Ocheltree, Alawi A Alsheikh-Ali, Abdul-Karim Al-Habib, Osama El-Sayed, Ali Haneef

**Affiliations:** 1 Section of Adult Cardiology, Cardiovascular Department, King Faisal Specialist Hospital & Research Center, Jeddah, Kingdom of Saudi Arabia; 2 Division of Cardiology, Baystate Medical Center, Tufts University School of Medicine, Springfield, Massachusetts; 3 Heart and Vascular Institute , Sheikh Khalifa Medical City, Abu Dhabi, United Arab Emirates; 4 Division of Cardiothoracic Surgery and Cardiac Surgery Intensive Care Unit, King Faisal Specialist Hospital and Research Center, Jeddah, Saudi Arabi

**Keywords:** Implantable cardioverter-defibrillator, radiofrequency ablation, coronary injury, pericardial effusion

## Abstract

All procedures have inherent risk. Our patient endured a sequence of rare life-threatening complications from commonly preformed procedures. The sequence of these complications was; large pericardial effusion post implantable cardioverter-defibrillator (ICD) implantation with echocardiographic signs of tamponade, left main narrowing post radiofrequency ablation, and late stent thrombosis post coronary intervention with a bare metal stent. All these occurred to one unfortunate young man. Furthermore, our patient demonstrated an unintended benefit of ICD which saved his life.

## Introduction

"Medicine is complex, medicine is unpredictable, and every patient is different" (Ignaz Semmelweis). Patients with cardiomyopathy due to frequent PVCs have limited treatment options. Anti-arrhythmic therapy has limited efficacy and a potential for multiple side effects including pro-arrhythmias. Catheter ablation is recommended when medication is not effective, tolerated, or desired, particularly in those with diminished systolic function. While catheter ablation can often be safely performed in close proximity to coronary arteries, risk of coronary injury can rarely occur. [1] Stent thrombosis is a serious adverse event after percutaneous coronary intervention. Our case describes a sequence of rare serious complications from the cardiac procedures performed and the unintended benefit of ICD.

## Case Report

A 19-year-old male patient presented to our cardiology clinic after a single unexplained episode of syncope. Seven months earlier, he was diagnosed with idiopathic dilated cardiomyopathy and he was started on evidence based therapy for his heart failure due to reduced left ventricular function. Diagnostic imaging studies performed on the patient at presentation are summarized in Table 1.

Given the risk of SCD, a decision was made to proceed with an ICD. He underwent Medtronic ICD implantation with a active-fixation atrial lead. On day one post ICD implantation, he developed pleuritic chest pain. Laboratory investigation revealed leukocytosis and elevated erythrocyte sedimentation rate (20 mm/Hr, reference range 0-5 mm/Hr). Chest X-ray showed properly positioned ICD leads in both atria and ventricles, without protrusion beyond the cardiac silhouette. Device interrogation was not suggestive of lead malposition given that both pacing and sensing thresholds did not stray from the initial intraoperative measures (both leads demonstrated minimal pacing threshold; sensing threshold was >2.5 mV in the atrium and >15 mV in the ventricle; impedance on both atrial and ventricular leads were within normal limits). Subsequent echocardiography revealed mild pericardial effusion. The patient was diagnosed as having device pericarditis and was kept under observation in our hospital. He was treated with high dose aspirin and colchicine and discharged 10 days later, after showing significant regression of his pericardial effusion by echocardiography.

**Figure 1:**
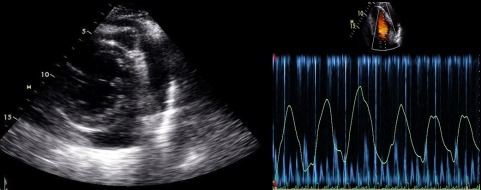
Parasternal short axis view (left panel) showing a circumferential pericardial effusion surrounding the left ventricle. Pulse wave Doppler (right panel) with respirometry of mitral inflow demonstrating the pronounced respiratory variation in filling.

 Our patient suffered a relapse 2 weeks following cessation of therapy and echocardiography demonstrated large pericardial effusion with echocardiographic signs of tamponade (Figure 1). He was re-hospitalized for the initiation of steroid therapy and the atrial lead was removed. Post lead removal, the patient remained clinically and hemodynamically stable with further regression of his pericardial effusion on subsequent echocardiography.

**Figure 2:**
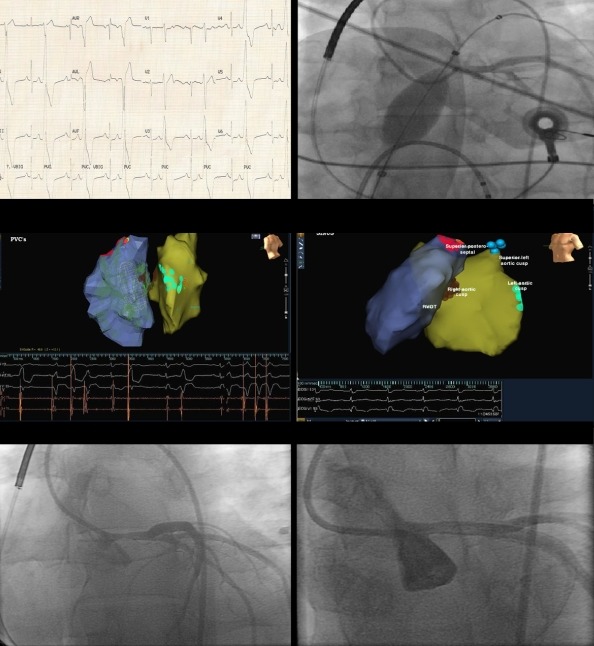
Upper left: 12-lead electrocardiogram (ECG) showing sinus rhythm with ventricular bigeminy. The ectopic beats show uniform left bundle branch block type morphology, inferior axis, and (rS) in V1-V2. Upper right: Diagnostic coronary angiogram showing normal coronaries and the position of the multi-electrode array catheter in the right ventricular outflow track (RVOT). Middle left: A 90 degree left lateral still image. Three ablation sites are displayed: the superior posteroseptal wall of the RVOT, and the right and left aortic cusps. Middle right: A 45 degree left anterior oblique still image showing the last ablation site along the superior border of the left aortic cusp. Lower left: Immediate coronary angiography post ablation at the last targeted site showing 80% left main narrowing. Lowr right: Coronary angiography post left main stenting showing good angiographic result.

Also during follow-up, given the large burden of PVCs (>25%) on Holter analysis the possibility that his cardiomyopathy might be reversible by PVC suppression was entertained, for which; a 6 month trial of amiodarone was started. Subsequent 24-Holter monitoring showed suppression of PVCs (≤4% of the total number of QRS complexes). Additionally, Echocardiography revealed significant improvement of his EF from 20% to 45%. Given the long term side effects of amiodarone therapy, particularly in young patients, the decision was made to stop amiodarone therapy and proceed with an electrophysiology study and radiofrequency ablation (RFA). At the family's behest, the patient was referred to a cardiac electrophysiology center in Europe with good experience in catheter ablation of PVCs. At base line sinus rhythm with ventricular bigeminy was recorded (Figure 2). A multi-electrode array catheter was positioned in the right ventricular outflow tract and a thermocool irrigated tip 7.5 Fr quadripolar ablation catheter with a 3.5-mm distal electrode, interelectrode spacing of 2-5-2 mm, and deflectable tip was used to create a 3D reconstruction of the right ventricular outflow track. Offline analysis of the ventricular ectopic beat showed an exit site at the superior posterior-septal wall with presence of small r-wave on the virtual unipolar signals. Ablation at this site failed to suppress the PVCs. Before moving the ablation catheter into the sinus of Valsalva via the retrograde aortic approach, an initial bolus of heparin was given followed by continuous infusion targeting activated clotting time of >250 seconds. Additionally, diagnostic angiography prior to left-sided mapping was performed, which demonstrated normal coronaries (Figure 2). A new 3D electro-anatomical mapping was created of the left ventricular outflow tract, using NavX. Ablation at the right and left aortic cusps, failed to suppress the PVCs (Figure 2). Subsequently, the ablation catheter was extended along the superior border of left aortic cusp, just below the left main ostium, where the best local activation time was recorded. Radiofrequency applications at this site (10 W, max 10 seconds) lead to immediate suppression of PVCs without recurrence (Figure 2).

The patient was asymptomatic with stable blood pressure and no ST changes. Because of the critical position of the last ablation site, he underwent immediate coronary angiography, which revealed significant (80%) left main narrowing. Intra-coronary nitroglycerin was given without response. On the same day, computed tomography scanning revealed no pathological finding of the ascending aorta and an echocardiography showed normal functioning aortic valve. On the following day, coronary angiogram was repeated, which was consistent with the previous angiographic findings. Subsequently, an intravascular ultrasound was performed and revealed cross-sectional area of 5.16 mm^2^. Thereafter, the patient underwent percutaneous coronary intervention with ballooning and stenting of the left main coronary artery, where a single Bare Metal Stent (BMS) (Multi-Link Vision by Abbott Vascular, 3.5mm x 8mm) was deployed. Post stent intravascular ultrasound revealed a 10.73 mm^2^ cross-sectional area with good stent wall opposition and expansion. Dual anti-platelet medication (aspirin and clopigrel) was initiated post-stenting. Subsequently, the patient was discharged.

**Figure 3:**
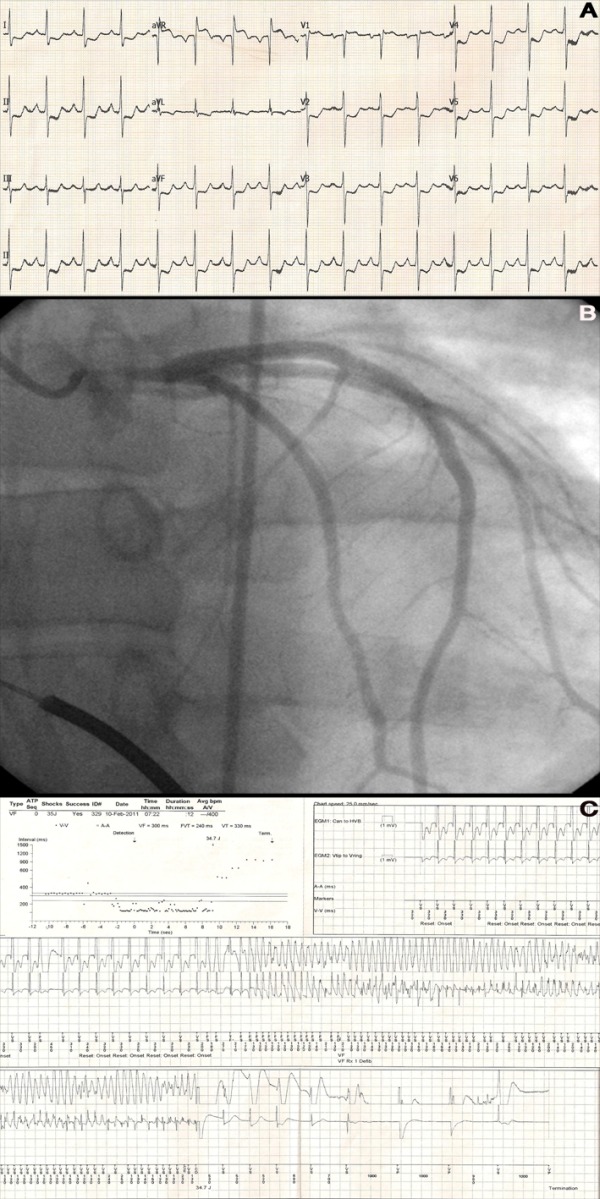
A: 12-lead electrocardiogram (ECG) demonstrating global ST depression B: Coronary angiography showing 95% left main stent thrombosis. C: Interval plot and stored electrogram showing appropriate device therapy due to ventricular arrhythmia (VF).

Four months later, the patient was brought to our emergency department by his family after suffering an episode of frank syncope. Upon clinical evaluation, he was asymptomatic and vitals were stable. Immediate 12-lead electrocardiography demonstrated global ST-segment depression; except in leads AVR and V1, which demonstrated ST-segment elevation (Figure 3). ICD interrogation revealed appropriate device therapy for ventricular fibrillation (Figure 3). An emergent coronary angiography revealed 95% left main stent thrombosis with Thrombolysis in Myocardial Infarction (TIMI) grade III flow (Figure 3). The patient was clinically stable and urgent coronary artery bypass surgery was performed. Finally, the patient was discharged and had a 6 month event-free follow-up. 

## Discussion

In this report, our patient endured ternary tribulations, which are considered rare and very serious complications to the procedures he underwent.

Incidence of device pericarditis ranged from 0% to 5% [2]. The etiology of pericardial effusion/tamponade in our patient was due to device pericarditis, not wall perforation [3]. The mechanism of device pericarditis is unclear, but it may involve direct irritation of the pericardium.[2] 

Incidence of coronary injuries during ablation procedures is low, <0.1% [1]. Different mechanisms have been proposed to explain acute coronary injury subsequent to RFA; they include spasm, dissection due to catheter manipulation, embolization secondary to thrombus formation at the site of RFA [1]. Direct thermal impact is the most likely mechanism in this case, which resulted in acute edema with wall thickening and luminal narrowing [4]. While coronary angiography was performed prior to left-sided mapping, it is possible that a repeat angiogram before the application of the last lesion may have alerted the operators to a prohibitive proximity of the lesion site to the left main origin.

One explanation for the late stent thrombosis could be due to the complete remission of the left main edema causing late strut malapposition which may increase the thrombotic risk due to the presence on intraluminal stent struts [5]. Intravascular ultrasound was not preformed to assess possible etiology of the late stent thrombosis due to our patient's acute presentation.

In our patient acute ischemic insult post stent thrombosis resulted in malignant ventricular arrhythmia; this life-threatening complication was appropriately treated by ICD. This was the first device therapy the patient had received since device implantation, which was both unintended and lifesaving. In retrospect, one could have suggested that the initial decision to implant an ICD in our patient was inappropriate as his cardiomyopathy proved to be reversible with suppression of PVCs. That "arguably" inappropriate implantation caused initial harm in the form of a rare complication of ICD implants, but later proved to be lifesaving in the face of another complication (stent thrombosis). The present case demonstrates the uncertainty and unpredictability of clinical medicine and its clinical application to a specific patient. In medicine we are taught Primum non nocere (above all else, do no harm) and as evident with our case there is an inherent risk to every procedure.

## Figures and Tables

**Table 1:**
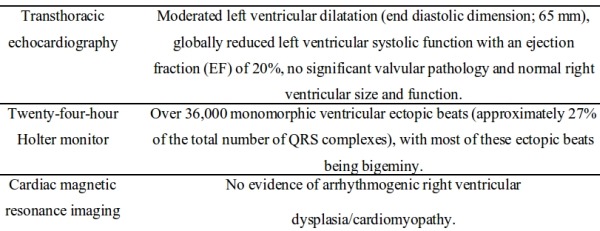
List of diagnostic imaging studies performed on the patient at presentation
